# Supplementary material for: Content-rich biological network constructed by mining PubMed abstracts
Source: BMC Bioinformatics. 2004 Oct 8;5:147. doi: 10.1186/1471-2105-5-147 (PMC528731; doi:10.1186/1471-2105-5-147)
Supplement: Additional File 2 — The original results of the above study (non-essential files are deleted to keep the file size under the limit set by BMC bioinformatics). [file 1471-2105-5-147-S2.bz2 › chilibotAdditionalFile2/dip10/387ID11297552E9861/html/PDGFA_PDGFRA.html]

 


 **PDGFA** and **PDGFRA** 
  
Found 8 abstracts in PubMed, retrieved 8.  
 

 What does Google say? 
 PDF only 
| .edu only 

---

**Interactive relationship** (e.g. stimulation, inhibition, etc)

**Neutral relationship**- The platelet derived growth factor receptor alpha  [ **PDGFRA** ]  subunit PDGFR alpha is the form of the PDGF receptor that is required for binding of PDGF A chain  [ **PDGFA** ] .  Ref: 1322269 Development, 1992

**Non-interactive relationship** (e.g. studied together, co-existance, homology, etc.)

- As expected, the fibroblasts produced primarily PDGF A chain  [ **PDGFA** ] , and the RLFs exhibited few PDGF alpha receptors PDGF R alpha  [ **PDGFRA** ] , the receptor type necessary for binding the AA isoform.  Ref: 9575867 Am J Physiol, 1998
